# *Dittrichia viscosa* L. Ethanolic Extract Based Ointment with Antiradical, Antioxidant, and Healing Wound Activities

**DOI:** 10.1155/2019/4081253

**Published:** 2019-04-22

**Authors:** Wafa Rhimi, Raoudha Hlel, Issam Ben Salem, Abdennacer Boulila, Ahmed Rejeb, Mouldi Saidi

**Affiliations:** ^1^Faculté des Sciences de Bizerte, Zarzouna, Université de Carthage, 7021, Tunisia; ^2^Laboratory of Biotechnology and Nuclear Technology, National Centre of Nuclear Science and Technology (CNSTN), Sidi Thabet Technopark, 2020 Ariana, Tunisia; ^3^Laboratory of Natural Substances LR10INRAP02, National Institute of Research and Physicochemical Analysis, Biotechpole of Sidi Thabet, Ariana 2020, Tunisia; ^4^Laboratory of Anatomy Pathology, University of Manouba, National School of Veterinary Medicine, Tunisia

## Abstract

*Dittrichia viscosa* which belongs to the* Asteraceae* family is frequently used to treat hematomas and skin disorders in Mediterranean herbal medicine. This study aims to validate its antioxidant effects and its potential on healing wounds. The ethanolic extract of* D. viscosa* leaves was formulated as 2.5% and 5% (w/w) in ointment bases on the beeswax and sesame oil. During this study, the ethanolic* D. viscosa* extract, ointments containing 2.5% and 5% of* D. viscosa *extract, and the vehiculum were assessed for their total phenol content (TPC), caffeoylquinic acid content (CQC), and antioxidant activities using complementary methods (TAC, the DPPH, ABTS, FRAP, and the BCB). The effects on wound healing of obtained ointments were evaluated by excision of the wound in a mice model for 12 days. Subsequently, the excised wound areas were measured at the 3^rd^, 9^th^, and 12^th^ days. The skin tissues were isolated for histological studies. The ointments containing* D. viscosa* extract (2.5%, 5%) possessed a considerable TPC, CQC, radical scavenging potential, and antioxidant activities compared to the vehiculum. Treated animals with ointments containing* D. viscosa* extract at 2.5% and 5% showed almost and totally healed wounds compared to the vehiculum and control groups, evidenced by good skin regeneration and reepithelialization. The present work showed the role of* D. viscosa* antioxidants exerted by its polyphenolic compounds, in particular, caffeoylquinic acids, in enhancing wound healing.

## 1. Introduction

The research to ensure a good quality of wound closure and scarless healing remains a health preoccupation until today [1, 2]. Recent investigation in wound healing mechanisms has evidenced that reactive oxygen species (i.e., hydroxyl OH^−^, peroxyl radicals ROO^−^, and superoxide anion O_2_^−^) act as mediator molecules between lymphoid cells and the wound sites as well as defensive molecules against pathogenic microorganisms in the wound area [3, 4]. In fact, basal level of reactive oxygen species (ROS) is necessary to regularize the inflammatory response, construction, and relaxation of blood vessels around wound areas [3, 4]. However, an excess of ROS level causes an imbalance between cellular production of free radicals (oxidants), and antioxidant defenses mechanisms, augmentation in inflammatory response, and inhibition of the wound repair [5, 6].

Indeed, antioxidants are scavengers molecules which are indispensable for neutralization of free radicals and for remediation of ROS damage during healing process [3, 6]. In this sense, plants extracts are emerging as a rich source of active compounds (i.e., triterpenes, flavonoids, polyphenolic, and tannins) for their pertinent properties to prevent from (i) accumulation of free radicals, (ii) oxidation of lipid, and (iii) inhibition of inflammatory disease. The use of several medicinal plants was strongly associated to their antioxidant properties. In particular,* Asteraceae *species plants were frequently used in wound healing treatment due to their high amount of phenolic compounds [7].

Among them,* Dittrichia viscosa* belonging to the genus of* Dittrichia* produced typical secondary metabolites such as phenolic compounds with antioxidant properties [7–10]. The* D. viscosa* was investigated against some free radicals (i.e., DPPH and ABTS). Then, some isolated flavonoids from this plant (i.e., sakuranetin, 7-O-methylaromadendrin, and 3-acetyl-7-O-methylaromadendrin) have been studied for their anti-inflammatory properties by subcutaneous injection of phospholipase A2 (PLA2) into mouse paws. However, to the best of our knowledge, the data about the antioxidant activities of crude extracts is scanty and there are no previous studies about the wound healing activity of* D. viscosa *ethanolic extract. The high use of this plant in traditional medicine in Mediterranean area explains our interest to suggest the usefulness of the extract of* D. viscosa* as wound healing ointment. The aims of the present study are to investigate in vitro the antiradical and antioxidant properties of ointments based on ethanolic* D. viscosa* extract and then to evaluate the potential of these ointments in wound healing in* vivo*.

## 2. Materials and Methods

### 2.1. Plant Extraction and Identification of Major Constituents

Fresh leaves of* D. viscosa* were collected from a remote area in Sidi Thabet, province of Ariana, North West of Tunisia. The plant was botanically identified in the Laboratory of Botany and Ornamental Plants, National Institute of Agronomic Research of Tunis. Leaves were air dried and then ground (0.5 mm) using blender mill. The powdered leaf was macerated in ethanol (10:100, w/v (g/ml)) during 48 h. After filtration, the solvent of extract was removed in rotary evaporator (Schwabach, Germany). The dried ethanolic extract was used for all experiments. The constituents of* Dittrichia viscosa* extract were identified using HPLC-DAD-ESI/MS as previously reported [11], and 20 *μ*L of extract at the concentration of 5 mg/mL was used for high performance liquid chromatography analysis (HPLC) using a chromatograph Alliance e2695 (waters, Bedford, MA, USA) equipped with photodiode array detector (PDA), interfaced with a triple quadruple mass spectrometer (MSD 3100, Waters) and an ESI ion source. The separation was carried out on an RP-xTerra MS column (150 × 4.6 mm. i.d., 3.5 *μ*m particles sizes). The phase mobile composed of water (A) and acetonitrile (B), both containing formic acid 0.1% with flow rate of 0.5 mL/min. The following gradient elution was used as follows: 0-40 min, 86 A%; 40-60 min, 85% A; 60-75 min, 100% A; 75-80 min, 86 % A. The mass spectra were acquired over m/z 100-1000 amu. The PDA acquisition wavelength was set in 200-800 nm, and the ionization conditions were performed as follows: electrospray voltage on negative mode of the ion source 25 V and a capillary temperature of 380°C. Mass Lynx v.4.1 software was used for data acquisition and processing. Identification of the constituents was based on their retention times, UV absorption spectra, and mass spectra data, as well as by comparison with authentic standards if available or literature data.

### 2.2. Ointment Preparation

#### 2.2.1. Formulation of Topical Preparation

Two concentrations of* D. viscosa* ethanolic extract (2.5% and 5% (w:w)) were used to formulate ointments according to the method of Alkafafy et al. [12] with a slight modification. Black sesame oil was heated to 100°C and ethanolic extract was added and homogenized for 3 min using an Ultra-Turrax homogenizer (T25, IKA Works, Wilmington, NC). Then, the liquefied beewax (10% of ointment) was added into the mixture and dispersed for 2 min using Ultra-Turrax homogenizer. Finally, obtained ointments were transferred to cool in ambient temperature and stored for all subsequent studies.

In order to evaluate the total phenol content, the caffeoylquinic acid content, and the antiradical and antioxidant potential of samples, ethanolic extract of* D. viscosa* was solubilized in methanol at a concentration of 1 mg/mL, while ointments containing* D. viscosa* (2.5% and 5%) and ointment base (vehiculum) were solubilized in dimethyl sulfoxide (DMSO) at concentration of 10 mg/mL.

#### 2.2.2. Total Phenol Content (TPC)

The TPC was determined using the Folin-Ciocalteu assay according to the method of Meda et al. [13]. Briefly, 500 *μ*L of each dissolved sample (extract, or ointment) was added to 2.5 mL of 10-fold diluted Folin-Ciocalteu reagent. Then, the 2 mL of saturated sodium carbonate (Na_2_CO_3_) solution (7.5%) was added to the mixture. The reaction mixtures were kept in the dark for 2 h. After the incubation, the samples absorbance was measured at 760 nm against the blank (methanol for extract and DMSO for ointments). All assays were conducted in triplicate and the results were averaged. The gallic acid (GAE) was used as the standard and the results were expressed as milligrams of gallic acid equivalent per gram of sample (extract or ointment) (mg GAE/g sample).

#### 2.2.3. Caffeoylquinic Acid Content (CQC)

The CQC in all samples was determined with the molybdate colorimetric assay according to the method of Chan et al. [14]. Briefly, 0.3 mL of appropriate sample solution was added to 2.7 mL of the molybdate reagent (1.65 g sodium molybdate, 0.8 g dipotassium hydrogen phosphate, and 0.79 g potassium dihydrogen phosphate in 100 mL of deionized water). The reaction mixture was incubated for 10 min, and its absorbance was measured at 370 nm against a blank sample. The chlorogenic acid (ChlA) was used as the standard and the results were expressed as milligram of ChlA equivalent per gram of sample (extract or ointment) (mg ChlA/g sample).

### 2.3. Antiradical and Antioxidant Properties

#### 2.3.1. Total Antioxidant Capacity (TAC) Method

The total antioxidant capacities of extract and ointments were evaluated using the phosphomolybdenum method described by Prieto et al. [15] with slight modifications. An aliquot of 0.25 mL sample solution (with concentration that ranged from 0.01 to 1mg/mL for extract and from 1mg to 10 mg/mL for ointments and vehiculum) was mixed with 0.75 mL of reagent solution (2.4 M of sulfuric acid, 112 mM of sodium phosphate, and 16 mM of ammonium molybdate). Methanol was used as blank. The tubes were capped and incubated in a boiling water bath at 95°C for 90 min. After the samples had cooled in room temperature, the absorbance of each sample was measured in spectrophotometer (Milton Roy, New York, USA) at 695 nm. Total antioxidant capacity was expressed as equivalents of ascorbic acid per gram of sample (extract or ointment) (mg AAE/g of sample).

#### 2.3.2. Diphenyl-1-Picrylhydrazyl (DPPH^•^) Method

The radical scavenging activity of the extracts against DPPH^•^ free radical was determined by the method of Molyneux et al. [16] with some adjustments. 1 mL of sample solution (with concentration that ranged from 0.01 to 1mg/mL for extract and from 1mg to 10 mg/mL for ointments and vehiculum) was combined with methanol DPPH^•^ solution (0.1 mM). The obtained samples were mixed vigorously and kept in the dark for 60 min.

Subsequently, the absorbance of each sample was measured at 517 nm. The scavenging activity was measured as the decrease in absorbance of the samples versus DPPH^•^ standard solution. BHT synthetic antioxidant was used as positive control. Results were expressed as radical scavenging activity percentage (%) of the DPPH^•^ according to the following equation:

(1) $$\begin{array}{c} \%\;\;DPPH\;\;radical\;\;scavenging=\frac{\left({Abs}_{0}-{Abs}_{s}\right)}{{Abs}_{0}\text{)}}*100 \end{array}$$

where Abs_0_ represents absorbance value of the control and Abs_s_ represents absorbance value of sample.

The DPPH radical scavenging activity is shown as EC_50_ (*μ*g sample/mL) which is the concentration necessary to 50% reduction of DPPH^•^ radical.

#### 2.3.3. 2-Azino-Bis-3-Ethylbenzothiazoline-6-Sulfonic Acid (ABTS) Method

The ABTS method is used according to Thaipong et al. [17] for investigating the radical scavenging capacity of each extract. The ABTS^•^ solution was prepared by the dissolving of 7 mM ABTS^•^ in deionized water with potassium persulfate (2.45 mM). The mixture was stranded in the dark at room temperature for 12-16 hours before use. The ABTS^•^ solution was diluted in methanol to an absorbance of 0.7 at 734 nm. For each analysis, a 0.15 mL aliquot of sample solution (with concentration that ranged from 0.01 to 1 mg/mL for extract and from 1mg to 10 mg/mL for ointments and vehiculum) was added to 2.875 mL of ABTS^•^ solution. The samples mixed were incubated for 15 min in the dark, and the absorbance was measured at 734 nm. BHT was used as standard.

Results were expressed in terms of EC_50_ (*μ*g sample/mL), which is the concentration necessary to 50% reduction of ABTS^•^ radical.

#### 2.3.4. Ferric Reducing Antioxidant Power (FRAP) Method

The FRAP assay was performed as described by the method of Gouveia et al. [18]. The stock solutions included 300 mM of acetate buffer (3.1 g of C_2_H_3_NaO_2_3H_2_O and 16 mL of C_2_H_4_O_2_, at pH 3.6, 10 mM of 2, 4, 6-tripyridyl-s-triazine (TPTZ) solution in 10 mM HCl, and 20 mM FeCl_3_ 6H_2_O solution). The FRAP solution was prepared by mixing acetate buffer, TPTZ solution, and FeCl_3_ 6H_2_O (10:1:1), and it was then warmed at 37°C before using. For each analysis, 0.15 mL of sample solution with concentration that ranged from 0.01 to 1mg/mL for extract and from 1mg to 10 mg/mL for ointments and vehiculum was added to 2,85 mL of the FRAP solution. The absorbance of the reaction mixture was measured at 593 nm after 30 min against methanol as blank. Results were expressed as micromole of Trolox equivalent per gram of sample (*μ*mol TE/g of sample).

#### 2.3.5. ß-Carotene Linoleic Acid (BCB) Method

The ß-carotene bleaching test was determined according to the method described by Velioglu et al. [19]. This assay based on the measure of the discoloration of ß-carotene during the oxidation of linoleic acid at 50°C of temperature. 0.2 mg of ß-carotene, 20 mg of linoleic acid, and 200 mg of tween 40 were dissolved in 0.5 mL of chloroform. After removing chloroform, 100 mL of oxygenated water was added to the final mixture and mixed until homogenization of the emulsion.

4 mL of the prepared mixture was added to 0.2 mL of sample solution (at concentration of 1mg/mL for extract and 10 mg/mL for ointments and vehicle) incubated for 2 h at 50°C in water bath. The BHT was used as a standard. The absorbance of all samples was measured at 470 nm at two times (t = 0 h and t = 2 h). The antioxidant power of sample was evaluated in terms of bleaching of ß-carotene using the following equation:

(2) %  inhibition  of  the  ß-carotene  bleaching  radical=100∗1−Abs0−AbstAbs0t=0−Abstt=0

where Abs_0_ (t=0) represents absorbance value of control at zero time, Abs_0_ represents absorbance value of control after 2 h of incubation, Abs_t_ (t=0) represents absorbance value of sample at zero time, and Abs_t_ represents absorbance value of sample after 2 h of incubation.

### 2.4. Wound Healing Experimental Design

Forty Swiss Webster mice weighing about 20-25g were purchased from the Pasteur Institute of Tunis, Tunisia. Animals were fed with standard pellet diet and were maintained under the following conditions: temperature (25 ± 3°C), humidity (60 ± 5%), and 24-h light/dark cycle. All experiments were performed with respect to the Institutional Animal Ethical Committee. Hair was shaved on the dorsal back of mice and disinfected with ethanol (70 %). Skin wounds of 10 mm diameter circular full-thickness were made on back of mice using a skin biopsy punch. Animals were randomly allocated into four groups (n=10 each): Group 1: negative control; the group had not received any healing creams/ointments. Group 2: the wounded area was treated with the ointment base (vehiculum). Group 3: the wounded area was treated with ointment of* D. viscosa* extract 2.5%. Group 4: the wounded area was treated with ointment of* D. viscosa* extract 5%. Ointments and vehiculum were applied daily during 12 post-wounding days [12]. At 3, 9, and 12 days of wound healing period, the wound area was measured and the percentage wound contraction was calculated according to the following equation:

(3) %  Wound  contraction=Wound  area  on  day"0"−Wound  area  on  day"n"Wound  area  on  day"0"×100

### 2.5. Histological Studies

On the 3rd, 9th, and 12th days, three mice were randomly taken from each group for the histological examination. The isolated wound tissues from mice skin were formalin fixed and paraffin blocked. Then, 5 *μ*m thick transverse incisions were made by means of a microtome fixed blade. Finally, sections were stained with hematoxylin and eosin (HE) and examined by light microscope (Olympus BX51) and photomicrographed using light microscope (Olympus BX51).

### 2.6. Statistical Analysis

Results were statistically analysed by using one way analysis of variance (ANOVA) test. Significant differences were set at p<0.05. The IBM SPSS 22 was used to perform statistical analysis.

## 3. Results and Discussion

### 3.1. HPLC-DAD-MS Analysis of* D. viscosa* Ethanolic Extract

Based on mass spectrum, UV spectra, and retention time of each peak of HPLC-PDA-ESI-MS/MS data, 29 phenolics were identified in ethanol extract of* D. viscosa *(Table 1).

The dicaffeoylquinic isomers (i.e., chlorogenic acid, 1, 3-O-dicaffeoylquinic acid, 3, 4 dicaffeoylquinic acid, 3, 5-dicaffeoylquinic acid, 1, 5-Di-caffeoylquinic acid, and 4, 5-dicaffeoylquinic acid) and quercetin derivatives (i.e., quercetin-galactosylrhamnoside, rutin-O-pentoside, quercetin-3-O-glucoside, rutin, and dihydroquercetin) were the major phenolic compounds. Others minor compounds (i.e., isoorientin, apigenin-glucoside, myricetin, and isorhamnetin-O-glucuronopyranoside) were also found.

To the best of our knowledge the triterpenoid ganoderic acids were identified for the first time in* D. viscosa* ethanolic extract. In this context, the comparison of our finding with previous results evidenced that the main representative compounds were unchangeable contrary to other minor compounds which are dependent on environmental factors (i.e., territory, temperature, and period of plant collection) and methods of extraction (materials, solvent, and extraction time) [11, 30].

### 3.2. Contents of Total Phenolics and Caffeoylquinic Acid

The TPC and CQC of* D. viscosa* ethanolic extract leaves, ointment base, and ointments containing 5% and 2.5% of extract have been reported in Table 2.

The TPC and CQC of* D. viscosa* ethanolic extract leaves were comparable to leaves of* Asteraceae* family plant (i.e.,* Cynara scolymus L.* and* Cynara cardunculus*) [31–33]. The TPC and CQC values of ointment containing 2.5% and 5% of* D. viscosa* ethanolic extract showed that there is no considerable loss in amount of both phenolic and caffeoylquinic acid compounds during the formulation of ointments. In this sense, it has been reported that the CQC are slightly modifiable after heating at temperature of 100°C for 5 min [34].

### 3.3. Antiradical and Antioxidant Properties

The TAC, The DPPH, ABTS, FRAP, and the BCB of* D. viscosa* ethanolic extract, ointment base, and ointment containing 5% and 2.5% of extract have been represented in Table 3.

The results of antiradical and antioxidants screening of* D. viscosa* leaves showed that our findings were comparable to other plants of* Asteraceae* family. In fact, the TAC, EC_50_ (DPPH), EC_50_ (ABTS), FRAP, and BCB values were in the range of other* Asteraceae* plants from different country where the TAC ranged from 110.03 to 194.64 mg AAE/g extract [35, 36], and EC_50_ (DPPH) values ranged from 100 to 250 *μ*g/mL [37]. EC_50_ (ABTS) ranged from 180 to 200 *μ*g/mL. FRAP values were ranging from 12.083 to 626.783 mg TE/g extract [38] and BCB percentage ranged from 34.8 to 75.20% [36, 39].

To the best of our knowledge there is no data on FRAP and BCB method of* D. viscosa *extracts. However, the TAC, EC_50_ (DPPH), and EC_50_ (ABTS) are in good agreement with those of Morocco* D. viscosa* leaves extracts [40]. Based on those findings the ethanolic* D. viscosa* exhibited a strong antioxidant activity explained by the high phenolic content, particularly by the highest caffeoylquinic acid content as indicated in Table 2. Previous investigation on plant of* Asteraceae* family was in relation to the role of polyphenols such as hydroxycinnamic acids (ferulic acid, p-coumaric acid, chlorogenic acid, and caffeic acid) on the antioxidant activity [41, 42]. Other studies confirmed the implication of dicaffeoylquinic derivatives in antioxidant activity and other biological activities [41, 43, 44].

In particular, the high contribution of caffeoyl derivatives in the antioxidant activities of* D. viscosa* was confirmed by Danino et al. [45] who proved that the isolated compound 1, 3-diCQA from* D. viscosa* has the greatest scavenging activity DPPH (EC_50_=40*μ*M) and ABTS (EC_50_ =12±0.4 *μ*M) than the trolox standard.

As shown in Table 3, 10 mg of ointments based on* D. viscosa* extract (2.5 and 5%) that exhibited excellent antiradical and antioxidant capacity values was comparable to 1 mg of BHT. The antioxidants, ointment samples showed dependence on to the concentration used. The ointment of* D. viscosa* extract (5%) possessed the high and the total antioxidant capacity comparing to ointment of* D. viscosa* extract (2.5%) and the base ointment, evidenced by its strongest radical scavenging activities, ferric reducing as well as BCB inhibition. These findings suggested that the formulation at temperature of 100°C did not affect significantly the phenolic composition and did not significantly change* the antioxidant activities*. Our findings were in agreement with previous studies that proved that phenolic acids (i.e., gallic, gentisic, protocatechuic, and caffeic acids) in pork lard showed a significant antioxidant activity at 150°C [46]. In another study, the addition of antioxidant (i.e., caffeic acid and tyrosol) into refined camellia oil before heating at temperature up to 120°C has been protecting the oil from oxidation and molecular changing [47].

### 3.4. Wound Contraction Ratio

**Figure 1:**
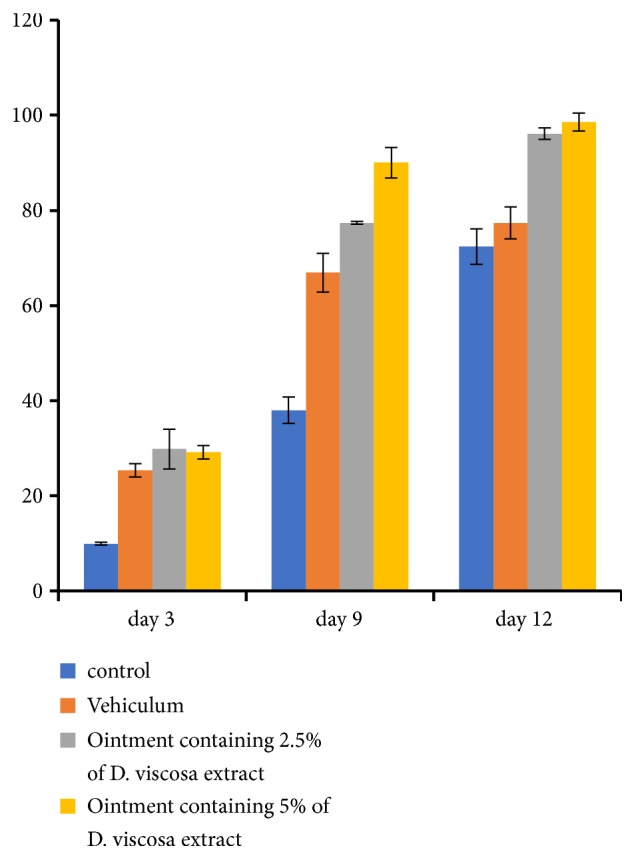
Percentage of wound contraction rate of mice treated with* D. viscosa* (2.5%, 5%) ointments, positive control (vehicle) and negative control on in vivo wound model.

**Figure 2:**
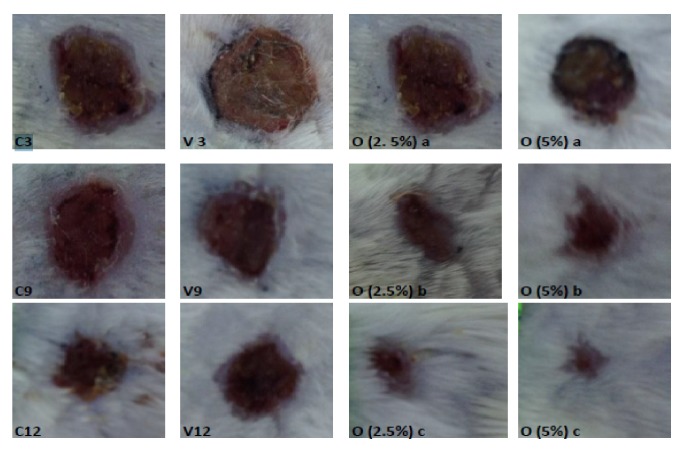
Morphological representation on wound contraction of different groups after 3, 9, and 12 days of topical application: *∗*C3: control day 3; *∗*C9: control day 9; *∗*C12: control day 12; *∗*V3: control day 3; *∗*V9: control day 9; *∗*V12: control day 12; O (2.5%) a: ointment containing* D. viscosa* (2.5%) day 3; O (2.5%) b: ointment containing* D. viscosa* (2.5%) day 9; O (2.5%) a: ointment containing* D. viscosa* (2.5%) day 12; O (2.5%) a: ointment containing* D. viscosa* (5%) day 3; O (2.5%) b: ointment containing* D. viscosa* (5%) day 9; O (2.5%) a: ointment containing* D. viscosa* (5%) day 12.

Comparing the three animal groups treated with ointments to the negative control, the wound area decreased significantly (p<0.05) by the twelfth day. The wound contraction ratio depends on the concentration of extract present in the ointment. In particular, on the 3, 9, and 12 days, the ointment containing* D. viscosa* 5% presented the highest wound contraction ratio to animals (Figures 1 and 2).

After 12 days, mice treated with ointments containing* D. viscosa* at 2.5% and 5% were totally healed while the vehicle showed a 57 % healing rate (Figure 2). A correlation between the antioxidant activities and wound contraction ratio of animals was observed. Thus, we suggest that antioxidant properties of* D. viscosa* enhanced the wound healing.

### 3.5. Histological Study

**Figure 3:**
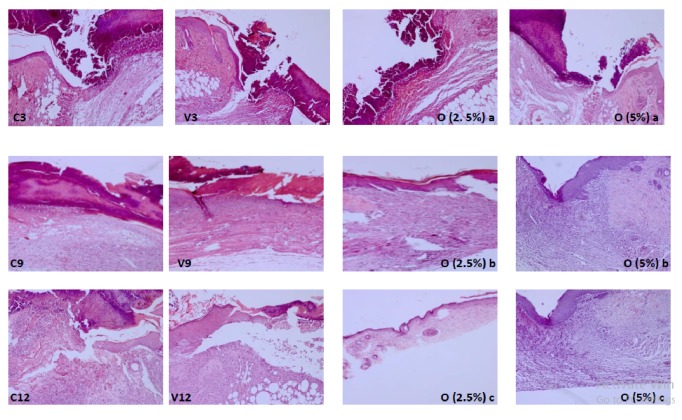
Histological sections of mice of different groups after 3, 9, and 12 days of topical application C3, V3, O (2.5%) a, and O (5%) a: complete destruction acute of epiderm, presence of inflammatory cells, and fibrin. C9, C12, V9, and V12: acute inflammation and destruction of epiderm. O (2.5%) b: proliferation of collagens fibers, inflammatory discreet infiltrate, and reepithelization. O (5%) b: proliferation of collagens fibers and inflammatory discreet infiltrate more important than those observed in O (2.5%) b. O (2.5%) c: Complete reepithelization. O (5%) c: reepithelization and crosslinking of collagen fibers.

Over 3 days, the tissue sections of all mice groups showed incomplete healing in wound site without significant difference between the groups; this was manifested by the large area of scab tissue. The inflammatory cells and fibrin were accumulated in granulation tissue and fibroblasts were dispersed (Figure 3: C3, V3, O (2.5%) a, and O (5%) a).

After 9 days, the tissue sections showed obvious difference between groups, though the number of inflammation cells decreased in all groups. Scab area tissue was observed only in the control group and vehicle group. On the other hand, there was great epithelial and collagen fibers organization, remarkable reduction in inflammation cell, and high distribution of fibroplasias. Groups treated with ointments containing* D. viscosa* (5%, 2.5%) showed greater healing quality compared with other groups manifested by tissue remodeling, the deposition of collagen in the wound and vessels regression, and mostly restored and keratinized epidermis after 9 days (Figure 3: O (5%) b and O (2.5%) b). With 5 % of* D. viscosa* extract, proliferation of collagens fibers was observed and inflammatory infiltrate was more important than those observed in group treated with 2.5% of* D. viscosa* extract (Figure 3: O (5%) b).

On day 12, the tissue section showed difference between untreated group (negative control, vehicle) and treated ones with ointments containing* D. viscosa*. Untreated group showed incomplete healing skin (Figure 3: C9, C12, V9, and V12), while group treated with ointment containing 2.5% revealed quasi-complete healing with maturated granulation tissue and hair follicles as well as highly organized collagen and high distribution of fibroblast cells (Figure 3: O (2.5%) c). Concerning the group treated with ointment containing 5% it showed complete healing and full reepithelialization where the numbers of cells and blood vessels were decreased significantly and the collagen fibers had been cross linked (Figure 3: O (5%) c).

In general, our findings are in agreement with previous observations that natural antioxidants promote the wound healing [12, 48, 49]. The wound healing is a dynamic interaction between epidermal and dermal cells and extracellular cells, which occurs in three successive phases: inflammation, proliferation, and maturation. In fact, during the inflammatory process the antioxidant altered the migration of the neutrophil to wound area and modulated neutrophil and macrophages influx (i.e., hydrolytic enzymes, reactive oxygen species, and reactive nitrogen species) [50], thus scavenging free radicals and preventing them from damage during proliferation and maturation process. The antioxidants stimulate synthesis of collagen, enhance cell proliferation and the angiogenesis, and promote the reepithelization of the wound [51]. In this context, some individual phenolic compounds (i.e., protocatechuic acid and caffeic acid) have a potential role in cytokines release in wound site, i.e., vascular endothelial growth factor (VEGF) and transforming growth factor beta (TGF-b) which are involved in remodeling the damaged tissue and accelerate the reepithelization [51, 52].

## 4. Conclusions

Our findings suggest that Tunisian* D. viscosa* could be a consistent source of antioxidant compounds particularly the caffeoylquinic, being able to scavenge free radicals and to prevent from oxidative damage. Subsequently, the investigation on properties of ointment containing* D. viscosa* leaves showed the potential antioxidant and wound healing effect. Thus, we suggest the role of phenolic compounds in antioxidant and healing wound activities. Hence, this study scientifically opens the perspective to the usefulness of* D. viscosa* as new pharmaceuticals product for oxidative stress and wound healing. However, further in* vivo* tests should be carried out.

## Figures and Tables

**Table 1:** Retention time, UV and mass spectral data, and tentative identification of the phenolic components in ethanolic leaves extractof *D. viscosa*.

Peak n	Tr (min)	*λ* max max	[M-H]^−^ (m/z)	Fragments ions (m/z)	Tentative of identification	Ref/std
1	7.969	325	353	191 (100)-161(10)	Chlorogenic acid	std
2	8.231	260-324	375	375(20)-191(100)	3-o-Caffeoylquinic acid	std
3	9.153	293-324-	543	387(50)-191(100)	1,3-O-Dicaffeoylquinic acid	std
4	11.405	260	599	467(100)	Ganoderic acid C_6_	[20]
5	11.694	260	599	467(100)	Ganoderic acid C_6_	[20]
6	12.243	280	583	467(100)-329(50)	Ganoderic acid D	[20]
7	18.712	284sh	609	429(60)-341(20)-301(100)-151(60)	Quercetin-galactosylrhamnoside	[21]
8	19.609	260-284sh	741	509(50)- 301(100)- 241(60)	Rutin-O-pentoside	[22]
9	20.751	260-331	463	301(100)-179(20)-151(30)	Quercetin-3-O-glucoside	[23]
10	21.158	260-332	591	301(100)-179(20)-151(30)	Rutin	std
11	22.791	326 sh	741	301(100)-241(20)-151(30)	Quercetin-7-O-xyloside-3-O-rutinoside	[23]
12	23.350	327	515	315(70)-191(40)-179(100)	3,4-Dicaffeoylquinic acid	std
13	25.543	327	515	353 (33)-191 (100)- 179(30)	3,5-Dicaffeoylquinic acid	std
14	26.253	327	677	515(15)-353(15)-191(100)-179(40)-135(20)	Dicaffeoylquinic acid glucoside	[24]
15	27.015	326	653	515(15)-353(15)-191(100)-179(40)-135(20	3, 4,5-Tricaffeoylquinic acid	std
16	28.901	327	515	353(30)-191(50)-179(100)-135(40)	1,5-Dicaffeoylquinic acid	std
17	29.180	327	515	353(30)-191(50)-179(100)-135(40)	3,5-Dicaffeoylquinic acid	std
18	30.525	324	591	509(30)-191(70)-179(100)	4,5-Dicaffeoylquinic acid	std
19	32.022	326	790	591- 405(80)-241(100)-191(60)	Dehydrodimers of caffeic acid	[25]
20	33.291	260-327	489	241(100)	Isoorientin	[26]
21	35.414	281-327	489	241(100)	Isoorientin	[26]
22	36.108	292-323	303	241(60), 151(100)	Dihydroquercetin	[21]
23	39.280	280-328	567	413(100)	Apigenin-glucoside	[26]
24	41.792	288	493	493(100)	Myricetin-O-glucuronide	[27]
25	44.651	332-288	495	493(100)	Dihydromyricetin-O-glucuronide	[28]
26	46.004	260-297-330	493	493(75)-315(100)-300 (50)-271 (80)	Isorhamnetin-O-glucuronopyranoside	[29]
27	47.815	260-290-331	533	515(100), 353 (80)	Dicaffeoylquinic derivatives	std
28	51.130	260-327	757	553(25), 323 (20), 203(100), 165 (50), 133 (30)	Caffeoyl-N-tryptophan	[26]
29	52.061	260-330	553	265(70), 203(100), 163(40)	Caffeoyl-N-tryptophan-rhamnoside	[26]

**Table 2:** Total phenol content (TPC), caffeoylquinic acid content (CQC) of *D. viscosa* ethanolic extract, ointment base, and ointment containing 5% and 2.5% of extract.

	Ethanolic extract [11]	Ointment containing 2.5% of extract	Ointment containing 5% of extract	Ointment base (vehiculum)
TPC (mg GAE/g of sample)	117.58 ± 1.29	4.70 ± 0.19	11.27 ± 0.121	0.94 ± 0.05
CQC (mg ChlA/g of sample)	71.85 ± 0.35	1.85 ± 0.06	4.77 ± 0.02	0.00±0.00

**Table 3:** Total antioxidant capacity, free radical scavenging (DPPH; ABTS), ferric reducing power, linoleic acid inhibition of *D. viscosa* ethanolic extract, ointment base, and ointment containing 5% and 2.5% of extract.

	*TAC* (mg AAE/g of sample)	*EC*_50_ *DPPH* (*μ*g/ml)	*EC*_50_ *ABTS* (*μ*g/ml)	*FRAP* (mg TE/g of sample)	ß-*carotene* *linoleic* *acid*
Ethanolic extract	133.02 ± 3.1	56.25 ± 1.2	147.26 ± 1.5	296.425 ± 3.3	54.01± 1.4
					(%I for 1 mg/mL)
Ointment base (Vehiculum)	1.61± 0.1	7977.00 ± 225.0	12550 ± 132	4.37 ±0.3	10.85 ± 1.1 (%I for 10 mg/mL)
Ointment containing 2.5% of extract	3.41 ±0.2	3073.70±138.8	6290± 183.9	11.69 ± 0.2	36.22 ± 0.9
					(%I for 10 mg/mL)
Ointment containing 5% of extract	7.46 ± 0.7	1360.50±90.6	3473.7± 217.5	19.85 ± 0.4	48.05± 1.8
					(%I for 10 mg/mL)
BHT	-	26.92 ± 1.22	42.64 ± 0.12	-	62.18 ± 1.6
					(% I for 1 mg/mL)

Values expressed are means ± S.D.

## Data Availability

The data used to support the findings of this study are available from the corresponding author upon request.

## Supplementary Materials

Supplementary Materials Graphic summary description: the ethanolic extract of* D. viscosa* leaves was leaves chemically characterized with HPLC-DAD-MS and it was used to formulate ointments (5% and 2.5% (w/w)) for healing wound test. Then, the antioxidants and the healing activities of those ointments were evaluated. The results obtained from this study revealed the excellent antioxidant potential of ointments base on ethanolic extract of* D. viscosa* and evidence their role in improving the rate and the quality of wound contraction samples.
