# Supplementary material for: Dittrichia viscosa L. Ethanolic Extract Based Ointment with Antiradical, Antioxidant, and Healing Wound Activities
Source: Biomed Res Int. 2019 Apr 22;2019:4081253. doi: 10.1155/2019/4081253 (PMC6501256; doi:10.1155/2019/4081253)
Supplement: Supplementary Materials — Graphic summary description: the ethanolic extract of D. viscosa leaves was leaves chemically characterized with HPLC-DAD-MS and it was used to formulate ointments (5% and 2.5% (w/w)) for healing wound test. Then, the antioxidants and the healing activities of those ointments were evaluated. The results obtained from this study revealed the excellent antioxidant potential of ointments base on ethanolic extract of D. viscosa and evidence their role in improving the rate and the quality of wound contraction samples. [file 4081253.f1.pptx]

## Slide 1
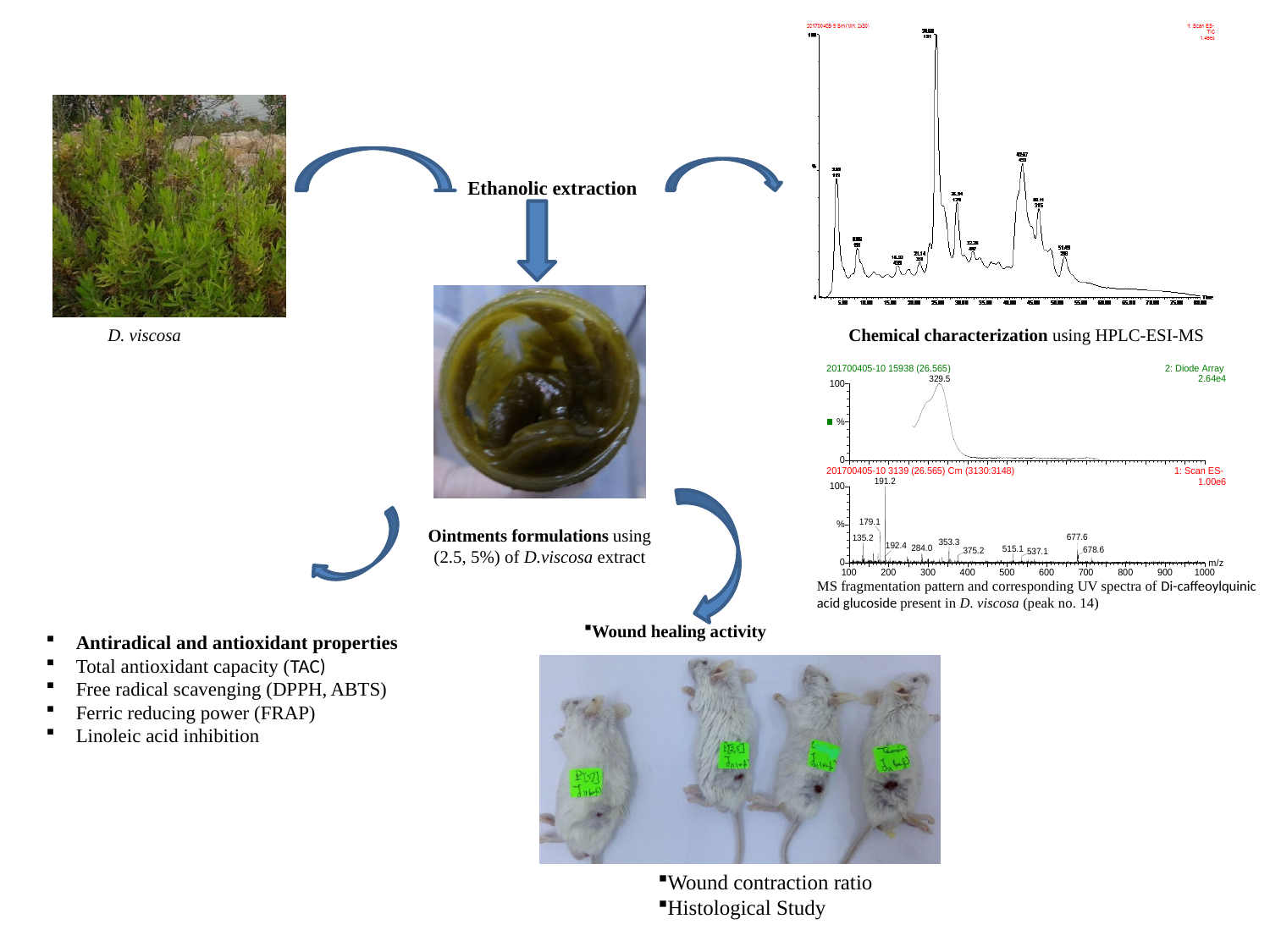

Ethanolic extraction
D. viscosa
Chemical characterization using HPLC-ESI-MS
Ointments formulations using (2.5, 5%) of D.viscosa extract
MS fragmentation pattern and corresponding UV spectra of Di-caffeoylquinic acid glucoside present in D. viscosa (peak no. 14)
Wound healing activity
Antiradical and antioxidant properties
Total antioxidant capacity (TAC)
Free radical scavenging (DPPH, ABTS)
Ferric reducing power (FRAP)
Linoleic acid inhibition
Wound contraction ratio
Histological Study
